# Optogenetic stimulation inhibits the self-renewal of mouse embryonic stem cells

**DOI:** 10.1186/s13578-019-0335-6

**Published:** 2019-09-03

**Authors:** Shaojun Wang, Lu Du, Guang-Hua Peng

**Affiliations:** 1 Department of Ophthalmology, General Hospital of Chinese People’s Liberation Army, Beijing, 100853 China; 2 Department of Ophthalmology, Affiliated Hospital of Academy of Military Medical Sciences, Beijing, 100071 China; 3 Department of Pathophysiology, Basic Medical College, Zhengzhou University, Zhengzhou, 450052 Henan China

**Keywords:** Optogenetic, Mouse embryonic stem cell, Proliferation, Self-renewal, Differentiation

## Abstract

Modulation of the embryonic stem cell state is beneficial for elucidating the innate mechanisms of development and regenerative medicine. Ion flux plays important roles in modulating the transition between stemness and differentiation in mouse embryonic stem cells (mESCs). Optogenetics is a novel tool for manipulating ion flux. To investigate the impact of optical stimulation on embryonic stem cells, optogenetically engineered V6.5 mESCs were used to measure the depolarization mediated by ChR2 on the proliferation, self-renewal, and differentiation of mESCs. Blue light stimulation significantly inhibited ChR2-GFP-V6.5 ESC proliferation and disrupted the cell cycle progression, reducing the proportion of cells in the S phase. Interestingly, optical stimulation could inhibit ChR2-GFP-V6.5 ESC self-renewal and trigger differentiation by activating the extracellular regulated protein kinase (ERK) signaling pathway. Our data suggest that membrane potential changes play pivotal roles in regulating the proliferation, self-renewal and initiation of differentiation of mESCs.

## Introduction

Mouse embryonic stem cells (mESCs) derived from the inner cell mass of blastocysts are considered multipluripotent with the characteristic of rapid self-renewal [1]. MESCs have been widely used to study embryonic development in vitro [2]. Clarifying the mechanisms for maintaining the ESC state or differentiation is beneficial for clinical applications. As far as we know, transcription factors (TFs) such as *Oct4*, *Sox2*, and *Nanog* form an autoregulatory network and act together to activate genes that maintain the pluripotent state and to silence genes for lineage-specific differentiation [3–7]. Many extracellular factors have been identified that trigger the transition of ESCs from self-renewal to differentiation by affecting the core TFs. However, the signaling pathways involved in this process require further investigation.

Ion channels contribute to the properties of cell membranes and play important roles in excitable as well as nonexcitable cells. Many factors can modulate the state of cell stemness by altering membrane properties. A previous study demonstrated that ESCs possess outward Kv currents, and when the K^+^ channels were blocked by TEA, mESC proliferation was significantly inhibited in a dose-dependent manner [8]. Furthermore, when the cell cycle was activated at G_0_ and progressed from G_0_ to S phase, the membrane potential changed regularly. Notably, membrane depolarization was able to modulate the proliferation process in dividing cells [9–11].

Optogenetics is a powerful technology that applies light and genetics to manipulate and monitor the activities of defined cell populations. Optogenetics has enabled great advancements in neuroscience [12, 13], and it has been utilized to examine the functional integration of neurons differentiated from ESCs in vivo by introducing the light-gated channelrhodopsin-2 (ChR2) into mESCs [14, 15]. Moreover, ChR2 has been found to enhance the differentiation of mESCs upon treatment with retinoic acid (RA), but the underlying mechanism is not clear [16]. The stem cell-based optogenetics approach provides an important tool for modulating the physiological state of stem cells. Thus, we examined whether the change in ion flux induced by blue light could modulate the ChR2-engineered-mESC fate from self-renewal to differentiation via the disruption of core TFs through the ERK signaling pathway.

## Materials and methods

### mESCs culture

V6.5 ESCs (derived from the F1 hybrid of 129SvJae/C57BL/6) were cultured on the dishes coated with 0.1% gelatin (Millipore, USA). The culture medium was prepared as preciously described [17].

### Creation of the ChR2-GFP-V6.5 ESC line

Lentiviruses containing the ChR2-green fluorescent protein (GFP) were packaged as previously, and the promoter ubiquitin C (UbC) controlled the expression of ChR2-GFP [18]. Viruses were concentrated and redissolved in phosphate-buffered saline (PBS). V6.5 ESCs cells were infected with the lentivirus (MOI = 5) with the addition of 5 μg/ml polybrene to enhance efficiency, followed by incubation for 6 h. After 1 week, the transduction efficiency was assayed via fluorescence microscopy. To obtain homogenous ChR2-GFP-V6.5 ESCs, cells were sorted via fluorescence activated cell sorting (FACS), and the top 5% of GFP-expressing cells were collected, and seeded one cell per-well in the 96-well plate. Subsequently, we harvested 13 colonies of the mESCs.

### Cell proliferation assay

A total of 1*10^5^ ChR2-GFP-V6.5 ESCs and noninfected V6.5 ESCs were seeded in a 35 mm dish coated with 0.1% gelatin. The cells in each dish were counted with a Coulter counter (Beckman Coulter, USA) after 2 days. Cells treated without optical stimulation and V6.5 ESCs were used as controls. The cell proliferation rate of both groups was calculated. The quantities of viable and nonviable cells were measured using the trypan blue exclusion assay.

### Immunostaining of cultured cell

ChR2-GFP-V6.5 ESCs seeded on Menzel-glass coverslips were fixed by 4% paraformaldehyde for 30 min and washed three times with PBS (5 min per wash). Then, the cells were permeabilized with 0.3% Triton and blocked with 10% bovine serum albumin (BSA) at 37 °C for 60 min and then covered with primary antibody solution and transferred to a cold room at 4 °C overnight. The cells were washed three times, and secondary goat anti-mouse antibody conjugated to Alexa Fluor 555 (Molecular Probe, USA) was added for incubation at 37 °C for 60 min. After the cells were washed three times with PBS, 4′,6-diamidino-2-phenylindole (DAPI) was added for 10 min, and the cells were washed in water three times and mounted using Fluoromount (Dako, Denmark). The primary antibodies were mouse anti-OCT4 (Chemicon, 1:200), Rabbit-anti SOX2 (Abcam, 1:200), Rabbit-anti NANOG (Abcam, 1:200), Mouse-anti Ki67 (Abcam, 1:200) and mouse anti-SSEA1 (Chemicon, 1:200). The images were acquired with a Leica TCS SP2 inverted confocal microscopy system with an HCX PL APO CS 40× 1.25 NA oil immersion objective (Leica, USA). Immunostaining semi-quantification between non-light and light stimulation groups was done as previous [19].

### Teratoma assay

For teratoma generation, the V6.5 ESCs or ChR2-GFP-V6.5 ESCs (5*10^6^) were injected subcutaneously into nude mice. The nude mice were fed and housed under a 12-h light–dark cycle. The animal protocol was approved by the Institutional Animal Care and Use Committee of the General Hospital of Chinese People’s Liberation Army and the Academy of Military Medical Sciences in accordance with the National Institutes of Health Guidelines for the Care and Use of Laboratory Animals. One group of mice was treated with optical stimulation at the injection sites every day (detailed in the optogenetic control section), while another group served as a nonphotoactivated control. After 30 days, tumors were harvested. Then, the size and weight of the teratomas from the photoactivated group were compared to those from the nonphotoactivated control group. All tissues were fixed in 10% formalin and embedded in paraffin, sections were stained with hematoxylin and eosin.

### Quantitative real-time PCR (qRT-PCR)

mRNA was extracted from ChR2-GFP-V6.5 mESCs with TRIzol on ice (Invitrogen, USA). First-strand cDNA synthesis was performed using the Thermo Script™ RT-PCR System (Invitrogen, USA). The mRNA level of each gene in both the photoactivated and nonphotoactivated control groups was determined by normalization to the *Gapdh* mRNA level. Real time-PCR was performed using SYBR Green Master Mix (Bio-Rad, USA) with a Bio-Rad system (Bio-Rad, USA) following the manufacturer’s instructions. The real-time PCR program was performed as follows: 40 cycles of denaturation at 95 °C for 10 s, annealing for 30 s, and elongation at 56–60 °C for 30 s. Nonspecific amplicons that appeared were evaluated by melting curves. All the primers corresponding to the examined genes are listed in Table 1.

**Table 1:** Mouse qRT-PCR primer sets

Gene	Forward sequence	Reverse sequence
*Oct4*	GTGGAGGAAGCCGACAACAATGA	CAAGCTGATTGGCGATGTGAG
*Sox2*	CAGGAGAACCCCAAGATGCACAA	AATCCGGGTGCTCCTTCATGTG
*Esrrb*	CAGGCAAGGATGACAGACG	GAGACAGCACGAAGGACTGC
*Klf4*	GTGCAGCTTGCAGCAGTAAC	AGCGAGTTGGAAAGGATAAAGTC
*Nanog*	TGGTCCCCACAGTTTGCCTAGTTC	CAGGTCTTCAGAGGAAGGGCGA
*Cdx2*	AGGCTGAGCCATGAGGAGTA	CGAGGTCCATAATTCCACTCA
*Gata6*	CTCAGGGGTAGGGGCATCA	GAGGACAGACTGACACCTATGTA
*Foxa2*	CCCTACGCCAACATGAACTCG	GTTCTGCCGGTAGAAAGGGA
*Fgf5*	CTGTATGGACCCACAGGGAGTAAC	ATTAAGCTCCTGGGTCGCAAG
*Nestin*	GCAGGGTCTACAGAGTCAGATCG	CAGCAGAGTCCTGTATGTAGCCA
*Gata2*	GCCTGTGGCCTCTACTACAAGC	CCCTTTCTTGCTCTTCTTGGAT
*Hand1*	AGGATGCACAAGCAGGTGA	GAGGCAGGAGGGAAGCTTT
*Brachyury*	GCTTCAAGGAGCTAACTAACGAG	CCAGCAAGAAAGAGTACATGGC
*Gapdh*	AGAGACGGCCGCATCTTCTTG	TGAAGGGGTCGTTGATGGCA

### Cell-cycle and apoptosis analysis

mESCs were trypsinized and fixed by 70% ice-cold ethanol. RNase A (25 mg/ml) was used to treat cells for 30 min at 37 °C to eliminate RNA. Cells were stained with 50 mg/ml propidium iodide for 10 min at room temperature load to FACSCalibur flow cytometer (BD Biosciences) to analysis cell cycle. Data acquisition was performed using CellQuest software (BD Pharmingen, USA), and the percentage of cells in the G_0_/G_1_, S, and G_2_/M phases was calculated using the Modfit. For analysis of apoptosis, each group of cells was washed with cold PBS, resuspended in 500 μl binding buffer, then transfer 100 µl to the tube and added 5 µl Annexin V-PE (Annexin V-PE apoptosis detection kit, BD Pharmingen, USA) and 5 µl 7-AAD, incubated in dark room at room temperature, subsequently added 400 µl binding buffer to the tube. Finally, the cells were analyzed using the FACS Calibur flow cytometer.

### Colony-forming assay

The colony-forming assay was performed by seeding 500 ChR2-GFP-V6.5 ESCs onto 35 mm dish and culturing with blue light, blue light + PD0325901, non-light, non-light + PD0325901 conditions, to form colony. After 7 days, cells were stained with Alkaline Phosphatase (AP) Detection Kit (Sigma-Aldrich, USA). AP-stained and differentiated colonies were counted to characterize the self-renewal ability of ChR2-GFP-V6.5 mESCs.

### Western blot analysis

For western-blot, protein was collect by lysing the cells with RIPA buffer on ice for 30 min. Then, 25 μg of lysate was added to SDS-PAGE and transferred to PVDF membrane (Millipore, USA). Membrane were blocked with 10% non-fat milk in PBS with 0.01% Tween 20 (1 h, 37 °C), then incubated with the first antibody overnight at 4 °C. First antibodies were used as the following dilutions: GAPDH (Abcam, USA) at 1:10,000, phospho-ERK (Abcam, USA) at 1:1000 and total ERK (Abcam, USA) at 1:2000. Then, washed three times in PBS/0.01% Tween 20, followed by incubating in secondary antibody (1 h, 37 °C). After washing three times, protein bands in the PVDF membrane was detected using an enhanced chemiluminescence (ECL) substrate (Pierce, Thermo Fisher Scientific, USA), and protein bands were visualized via film exposure. GAPDH expression was used as an internal control. The protein expression level was determined by band density with Quantity One software (Bio-Rad, USA).

### Electrophysiology

Whole-cell-patch clamp recording of cultured ChR2-GFP-V6.5 ESCs was performed in a dark room at 26 °C. ESCs were seeded onto Germany-glass coverslips in the dish. Cells were continuously perfused with external solution composed of (in mM) 145 NaCl, 5 KCl, 1.8 CaCl_2_, 1 MgCl_2_, 10 HEPES and 10 glucose at pH 7.4, and an osmolarity of 290–310 mOsm. The photoactivation-induced inward currents were recorded using an Axopatch 200B patch amplifier (Axon Instruments, USA) and digitized using a Digidata 1440 A/D converter and Clampex 10.0 software (Molecular Devices, USA). Borosilicate glass tubing was applied to prepare recording pipettes by using a horizontal puller (P-97, Sutter Instruments, USA); typically, the recording pipettes exhibited a resistance of 3.0–5.0 MΩ when filled with pipette solution containing (in mM) 140 KCl, 10 EGTA, 10 HEPES and 5 MgATP at pH 7.3, and an osmolarity of 290–310 mOsm. After establishment of the whole-cell configuration, the adjustments to compensate for capacitance and series resistance were performed prior to recording the membrane signals. Between 70 and 80% of the series resistance was compensated electronically. Signals were filtered at 2 kHz and digitized at 10 kHz. A leak subtraction protocol was performed. 470 nm blue light was delivered using a 40× water immersion microscope objective and a digitally operated shutter.

### Calcium imaging

Calcium indicator Rhod-2 (Molecular probe, USA) were diluted into filtered recording buffer (NaCl 129 mM, KCl 4 mM, MgCl_2_ 1 mM, CaCl_2_ 2 mM, Glucose 10 mM, HEPES 10 mM) to obtain a loading solution with a final concentration in calcium indicator of 1 μg/ml. The culture medium was replaced with loading solution and the cultures were incubated at room temperature for 20 min in the dark. The loading solution was then removed and replaced by fresh recording buffer, and the cultures were allowed to recover at 37 °C for 30 min in the dark before imaging. The cells were stimulated with blue light (470 nm) for 5 min (light intensity 10 mW/mm^2^). We also added the KCl (5 mM final concentration) in recording buffer to induce the membrane depolarization and recording the calcium waves. In addition, for distinguishing the calcium source, we applied recording buffer without calcium. Images were acquired with a scanning confocal microscope (Leica, USA) and analyzed by ImageJ.

### Optogenetic control

The cells were cultured in the room without light. Light stimulation was applied with a 470 nm light-emitting diode (LED) device (Thorlabs, USA). For whole-cell patch-clamp recording, a digitally controlled 470 nm LED light was projected to the back of a 40× water immersion microscope objective. The light intensity was modulated using a potentiometer and ranged from 0.1 to 10 mW/mm^2^. The inward current peak was calculated for different light intensities. For optogenetic stimulation of cultured ChR2-GFP-V6.5 ESCs, optical stimulation was conducted in the incubator using the LED light source. All regions of the culture plate were stimulated with blue light (470 nm) for 5 min/h (10 ms with 90 ms intervals and 10 Hz flickering light, 5 min/h duration, light intensity 10 mW/mm^2^). When the stimulation protocol was finished, the cells were fixed on the Menzel coverslips, and sister cells were prepared for immunostaining, qRT-PCR and cell counting. Additionally, we prepared cells exposed to different light intensities (low intensity: 0.1 mW, medium intensity: 1 mW, high intensity: 10 mW) for Western blot analysis. For analyzing the role of ERK, we have added PD0325910 to the culture medium, then the cell underwent optical stimulation. For optical stimulation during teratoma assays, all of the animals were anesthetized (4% chloral hydrate) every day after cell injection at the same time, and we choose the same light intensity used for the cultured cells for the injected cells (10 Hz, 1 h duration, 10 mW/mm^2^, beneath the skin at the injection site, actual value of 70 mW/mm^2^ on the surface of the skin). The light stimulation was delivered by applying an LED light source to the injection site skin. Nonphotoactivated control animals were treated as described above without optical stimulation.

### Statistical analysis

All data are presented as the mean ± SD. Student’s t-test was performed to compare the differences between groups. And the expression of ERK was analyzed by one-way analysis of variance (ANOVA). P < 0.05 was considered statistically significant.

## Results

### Characterization of ChR2-GFP-V6.5 ESCs

**Fig. 1:**
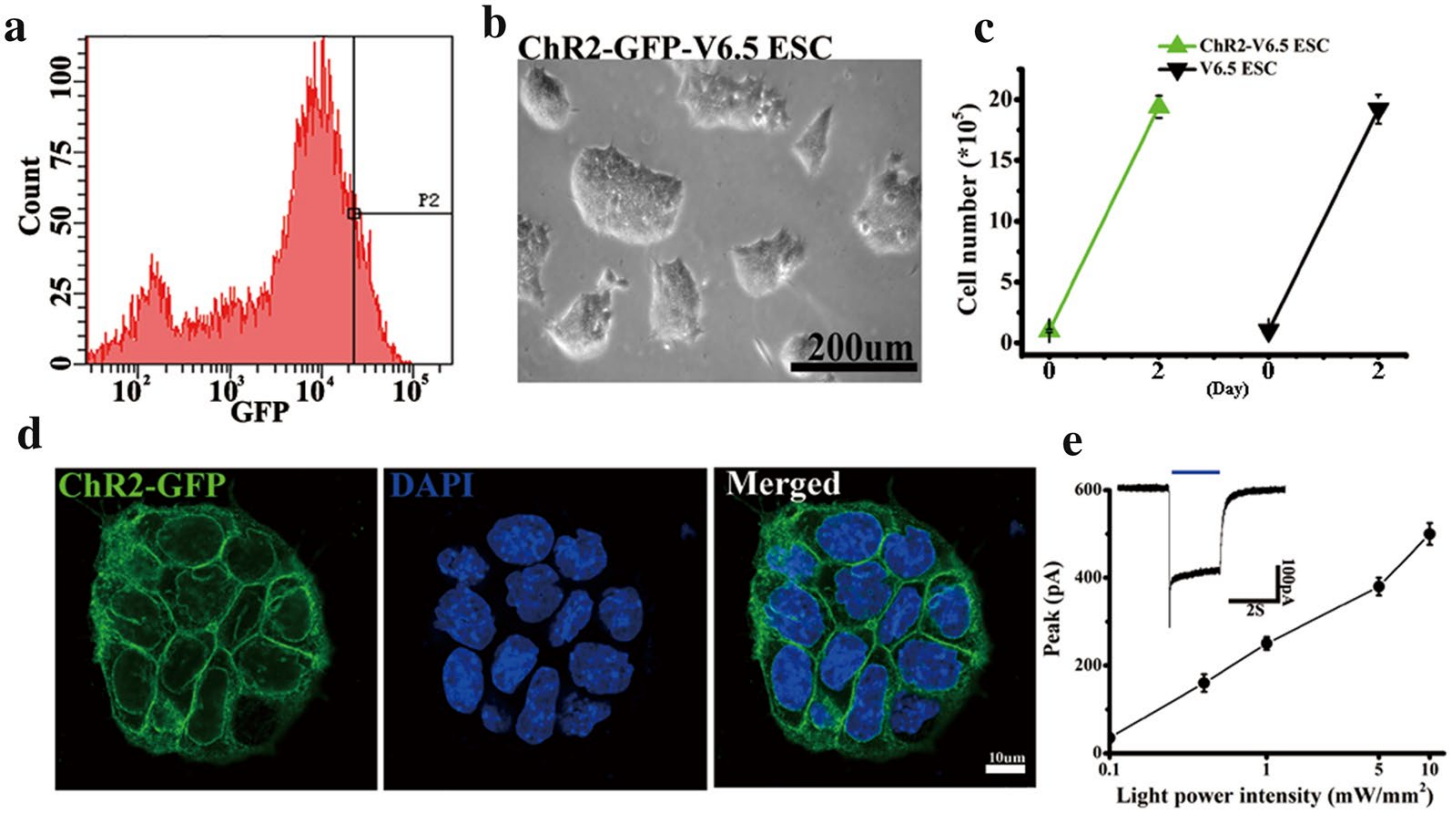
Functional expression of ChR2 in V6.5 ESCs. **a** FACS sorting based on the GFP expression in the cell population. A gate for the 5% of cells with the most fluorescence was selected. **b** Brightfield image of ChR2-GFP-V6.5 ESCs after FACS sorting. **c** Cell proliferation assay of V6.5 ESCs and ChR2-GFP-V6.5 ESCs. **d** Laser confocal scanning of ChR2-GFP-V6.5 ESCs revealing ChR2-GFP in the membrane. **e** Electrophysiological recording of light (wavelength of 470 nm) of various intensities stimulating inward currents in ChR2-GFP-V6.5 ESCs (the blue bar represents the light stimulation). Inset, representative ChR2 photocurrents at a light intensity of 5 mW (n = 6, *P < 0.05 compared to the wild-type control)

**Fig. 2:**
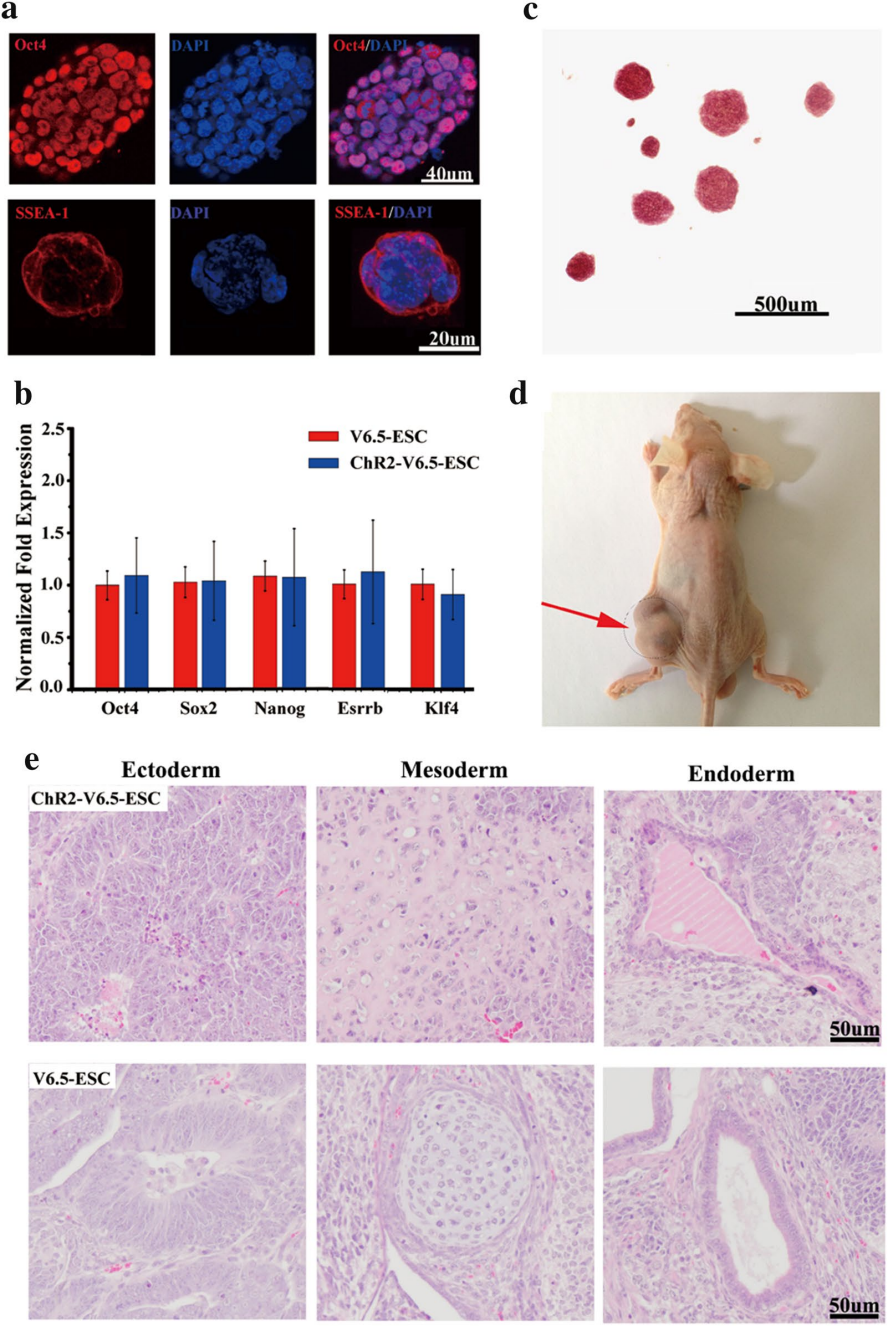
Characteristics of the ChR2-GFP-V6.5 ESCs. **a** Immunostaining for the pluripotency markers Oct4 and SSEA1 in ChR2-GFP-V6.5 ESCs. **b** qRT-PCR analysis of the pluripotency-associated genes *Oct4, Sox2*, *Nanog*, *Esrrb* and *Klf4* in ChR2-GFP-V6.5 ESCs and V6.5 ESCs. **c** AP staining of ChR2-GFP-V6.5 ESCs. **d** A nude mouse subcutaneously injected with 5*10^6^ ChR2-GFP- V6.5 ESCs on the left side formed a teratoma. (n = 6, *P < 0.05 compared to the wild-type control). **e** Hematoxylin and eosin staining of teratomas derived from V6.5 ESCs and ChR2-GFP-V6.5 ESCs

First, V6.5 ESCs were infected with a lentiviral ChR2-GFP driver by the UbC promoter. On the basis of the expression level of GFP, the top 5% cells were selected by FACS (Fig. 1a). The colony morphology and proliferation rate of the ChR2-GFP-V6.5 ESCs have no significant difference from those of nontransduced V6.5 mESCs (Fig. 1b, c) (n = 6, P > 0.05 vs. control). We found that the ChR2-GFP protein localized in the ESC membrane by using laser confocal scanning microscopy (Fig. 1d). Blue light (470 nm) generated inward photocurrents (470 nm, 2 s pulse duration) in an intensity-dependent manner in ChR2-GFP-V6.5 ESCs, while the steady-state photocurrents showed little inactivation (Fig. 1e). Therefore, the inward currents induced by blue light led to membrane depolarization. ChR2-GFP-V6.5 ESCs expressed the ESC markers OCT4 and SSEA-1, as indicated by immunostaining (Fig. 2a), and the core TFs, including *Oct4*, *Sox2*, *Nanog*, *Esrrb* and *Klf4*, as measured via qRT-PCR (Fig. 2b) (n = 6, P > 0.05 vs. control), indicating that ChR2-GFP-V6.5 ESCs, just like noninfected cells, are in an undifferentiated state. Additionally, all of the ChR2-GFP-V6.5 ESCs were positively stained for alkaline phosphatase (AP) (Fig. 2c). Teratomas were also detected after 30 days when the ChR2-GFP-V6.5 ESCs were subcutaneously injected into nude mice (Fig. 2d). And the teratomas between V6.5 ESCs and ChR2-GFP V6.5 ESCs has no obvious difference (Fig. 2e). Together, these results indicate that the introduction of ChR2 into V6.5 ESCs does not modulate the stemness of these cells.

### Optical stimulation inhibited the proliferation and disrupted the cell cycle progression of ChR2-GFP-V6.5 ESCs

**Fig. 3:**
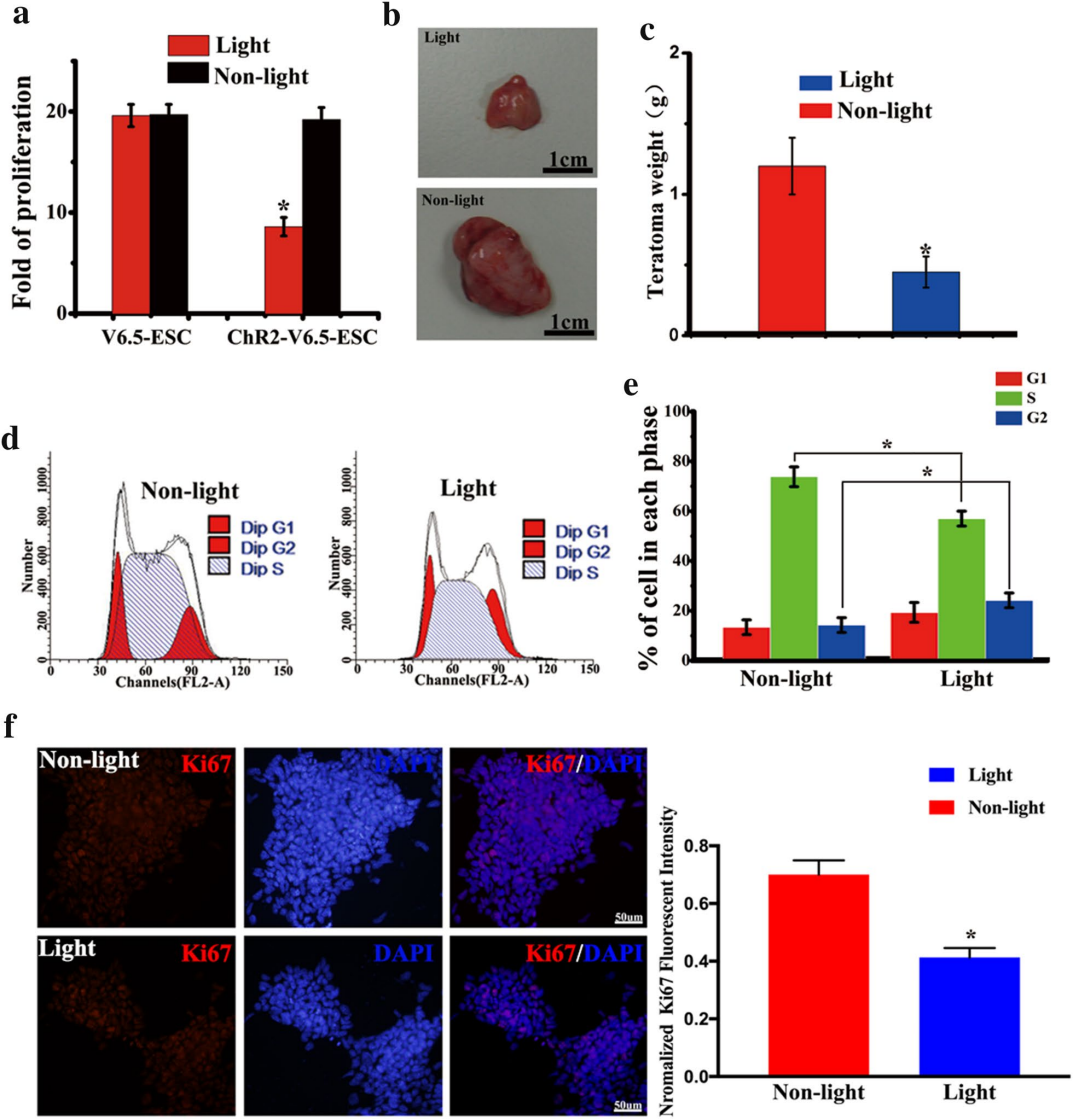
Photoactivation inhibits the proliferation of ChR2-GFP-V6.5 ESCs. **a** Cell proliferation assay of V6.5 ESCs and ChR2-GFP-V6.5 ESCs in both the photoactivated group and the nonphotoactivated control group. **b**, **c** Size and weight analysis of the teratoma formed by ChR2-GFP-V6.5 ESCs in both the photoactivated group and the nonphotoactivated control group. **d**, **e** Flow cytometric analysis of the cell cycle distribution of ChR2-GFP-V6.5 ESCs. (n = 6, *P < 0.05, **P < 0.01, ***P < 0.001 compared to the nonphotoactivated control). **f** Immunostaining and semi-quantification assay of Ki67 expression in both the photoactivated group and the nonphotoactivated control group. (n = 6, *P < 0.05 compared to nonphotoactivated control)

**Fig. 4:**
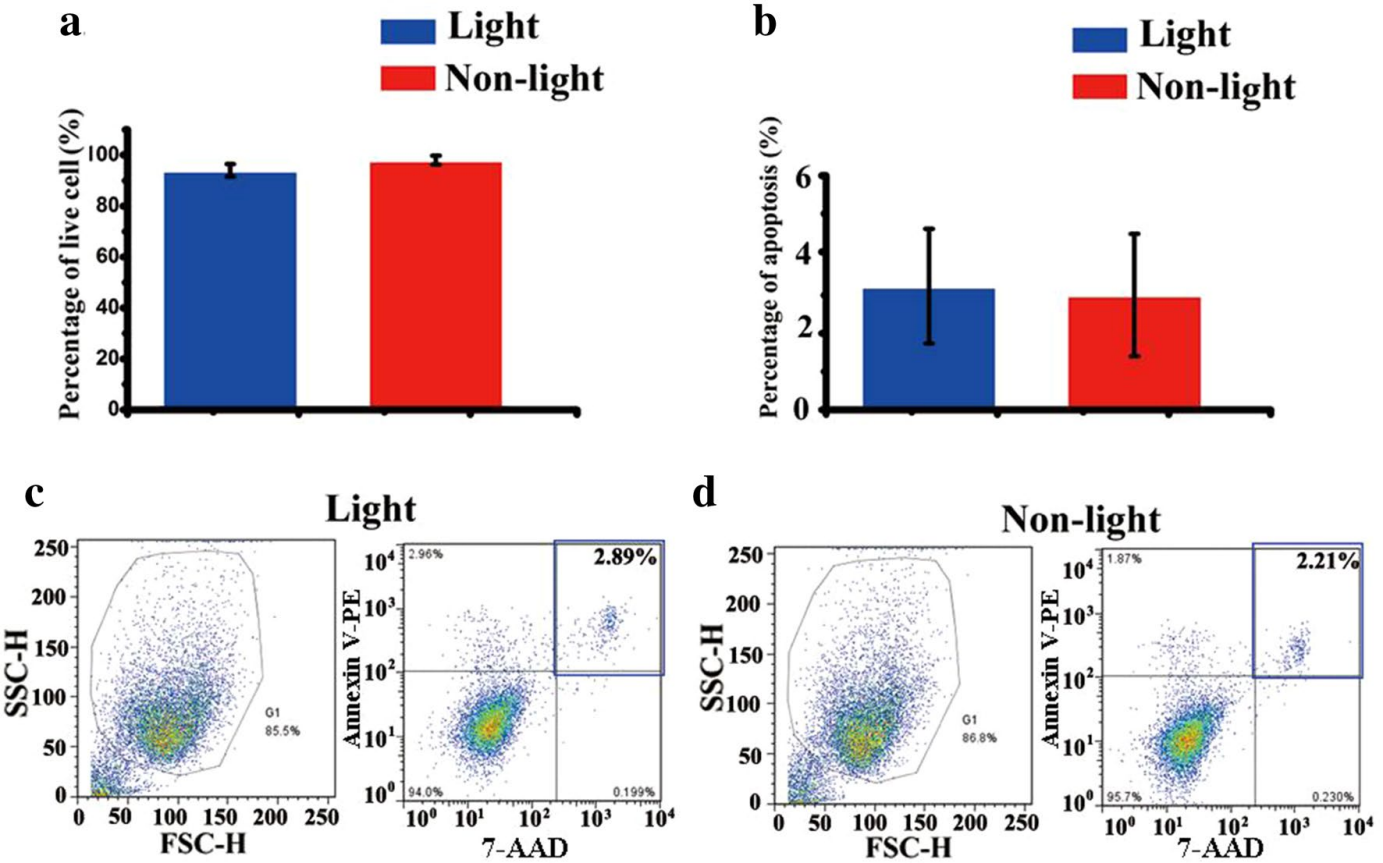
Optical stimulation does not alter the viability of ChR2-GFP-V6.5 ESCs. **a** The results of the cell viability assay of ChR2-GFP-V6.5 ESCs (trypan blue exclusion assay) in both the photoactivated group and the nonphotoactivated control group are summarized in a bar graph. **b** Summary graph of the apoptosis of ChR2-GFP-V6.5 ESCs in both the photoactivated group and the nonphotoactivated control group. **c**, **d** Flow cytometry quantifying the apoptosis of ChR2-GFP-V6.5 ESCs in the nonphotoactivated and photoactivated groups. (n = 6, *P < 0.05 compared to the nonphotoactivated control)

Subsequently, we examined whether blue light-induced membrane depolarization would affect the proliferation of ChR2-GFP-V6.5 ESCs. The application of blue light stimulation for 48 h inhibited the proliferation of ChR2-GFP-V6.5 ESCs, as indicated by the number of cells compared to that of control ESCs (n = 6, P < 0.05 vs. control), while optical stimulation had no impact on the proliferation of wild-type V6.5 ESCs (Fig. 3a) (n = 6, P > 0.05 vs. control). The ChR2-GFP-V6.5 ESCs were injected under the nude mouse skin, 470 nm blue light stimulation was performed every day. The size of teratomas was much smaller in 470 nm blue light stimulation group compared with that in the nonphotoactivated control group (Fig. 3b, c) (n = 6, P < 0.05 vs. control). Optical stimulation reduced the percentage of cells in the S phase while increasing that in the G_2_ phase (Fig. 3d). The proportion of cells in the S phase decreased from 73.75 ± 4% in the control group to 56.75 ± 2.8% in the photoactivated group (n = 6, P < 0.05 vs. control). The percentage of cells in the G_2_ phase in the nonphotoactivated control group was 13.57 ± 2.5%, while photoactivation increased the percentage of cells in the G_2_ phase to 23.57 ± 2.9% (n = 6, P < 0.05 vs. control). The cell cycle characteristics of the photoactivated ChR2-GFP-V6.5 ESCs are summarized in Fig. 3e. The expression of Ki67 obviously decreased with blue light stimulation (Fig. 3f). Furthermore, to determine whether photoactivation could affect the viability of ChR2-GFP-V6.5 ESCs, we measured cell viability by the trypan blue assay. Photoactivation did not affect the viability of ChR2-GFP-V6.5 ESCs (Fig. 4a) (n = 6, P > 0.05 vs. control). We then used flow cytometry to measure the apoptosis rates of the photoactivated group and the nonphotoactivated control group, and no notable difference was found between these two groups (Fig. 4b–d) (n = 6, P > 0.05 vs. control).

### Optical stimulation inhibited the self-renewal of ChR2-GFP-V6.5 ESCs

**Fig. 5:**
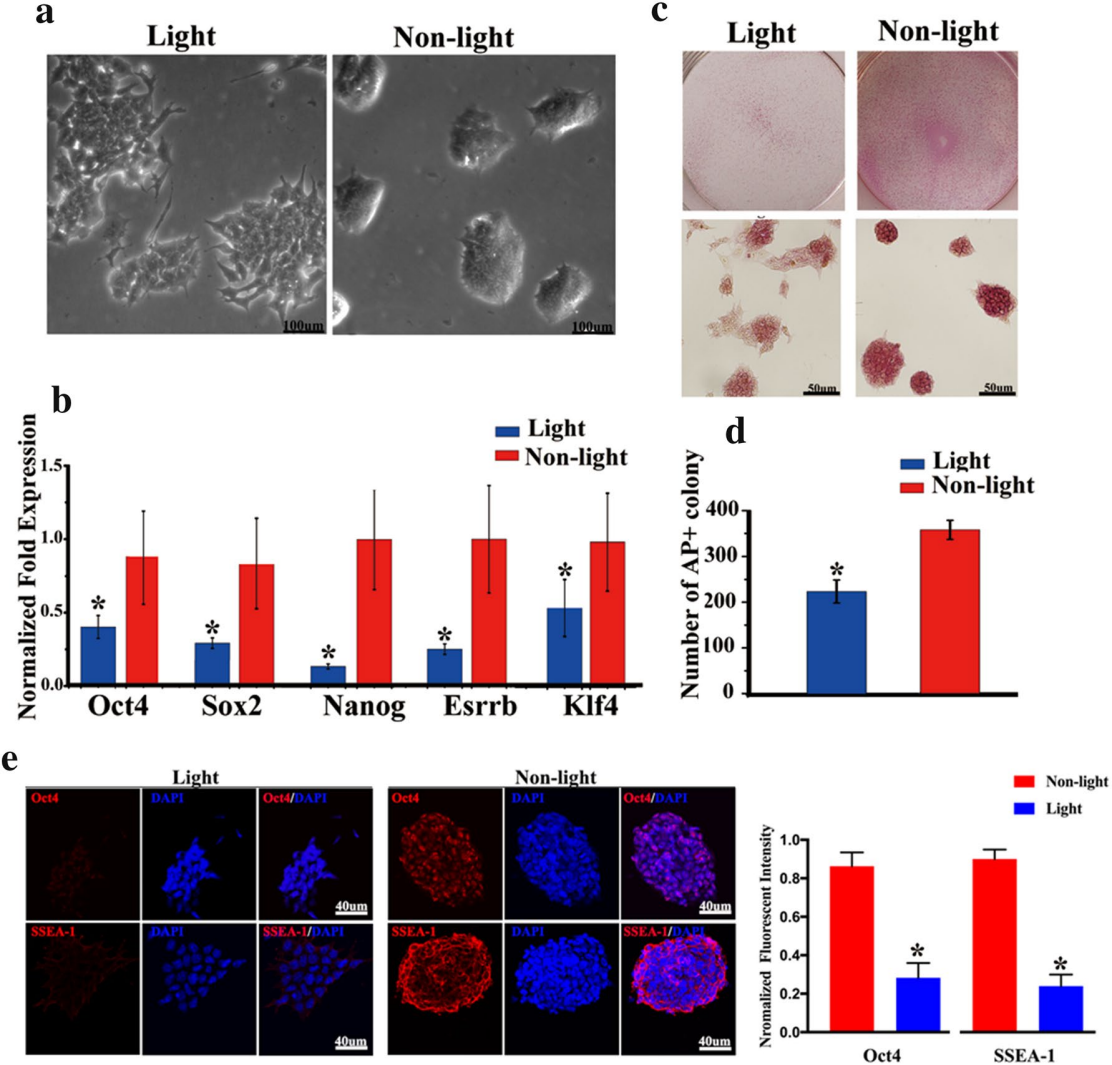
Photoactivation inhibits the self-renewal of ChR2-GFP-V6.5 ESCs. **a** Brightfield image of photoactivated and nonphotoactivated ChR2-GFP-V6.5 ESCs. **b** qRT-PCR analysis of the TFs *Oct4*, *Sox2*, *Nanog*, *Esrrb* and *Klf4* in photoactivated and nonphotoactivated ChR2-GFP-V6.5 ESCs. **c** Colony formation assay of photoactivated and nonphotoactivated ChR2-GFP-V6.5 ESCs. **d** Statistical analysis of the colony formation data of photoactivated and nonphotoactivated ChR2-GFP-V6.5 ESCs. **e** Immunostaining and semi-quantification for the pluripotency markers OCT4 and SSEA-1 in photoactivated and nonphotoactivated ChR2-GFP-V6.5 ESCs. (n = 6, ***P < 0.001 compared to the nonphotoactivated control)

In addition to having a slower proliferation rate, photoactivated ChR2-GFP-V6.5 ESCs displayed flatter colony morphology than the nonphotoactivated control ChR2-GFP-V6.5 ESCs (Fig. 5a). Because the core TFs are essential for ESC self-renewal, we measured the mRNA levels of *Oct4*, *Sox2*, *Nanog*, *Esrrb* and *Klf4* by qRT-PCR. We found significant downregulation of these pluripotency markers in the cells of the photoactivated group compared to those of the nonphotoactivated group (Fig. 5b) (n = 6, P < 0.05 vs. control). Moreover, the immunostaining has shown the decrease of OCT4 and SSEA-1 in the nonphotoactivated group [(Fig. 5e) (n = 6, P < 0.05 vs. control)]. To determine whether reduced core TF activity after optical stimulation is involved in the compromised self-renewal, we examined the ability of ChR2-GFP-V6.5 ESCs to form colonies upon optical stimulation. Single cells were seeded and treated with or without 7 days of light exposure. The colonies formed from a single cell were stained for AP (Fig. 5c). ChR2-GFP-V6.5 ESCs without light exposure developed numerous AP-positive colonies, while photoactivation significantly reduced the number of AP-positive colonies (Fig. 5d) (n = 6, P < 0.05 vs. control).

### Optical stimulation triggers the differentiation of ChR2-GFP-V6.5 ESCs via ERK activation

**Fig. 6:**
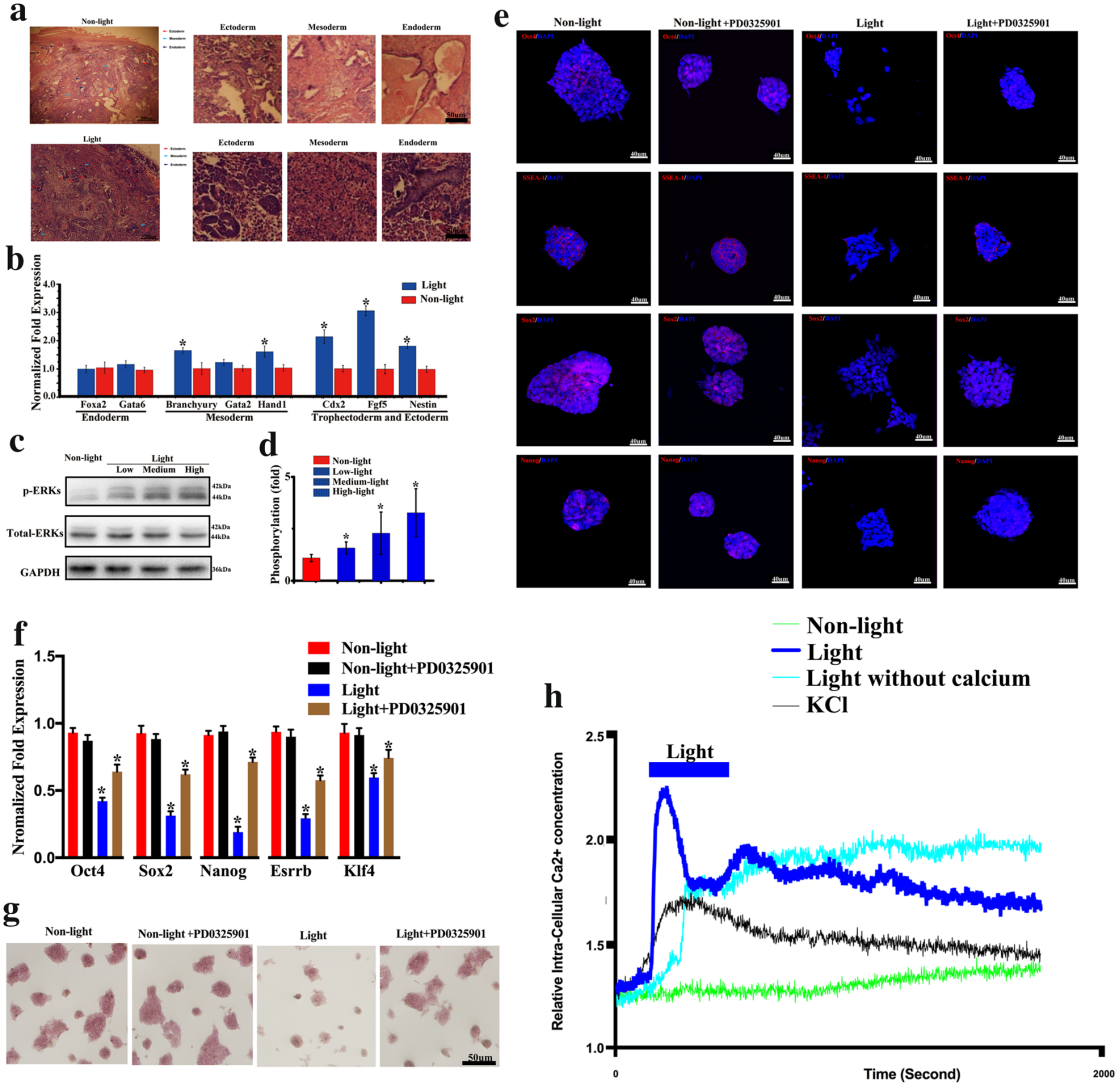
Photoactivation triggers the differentiation of ChR2-GFP-V6.5 ESCs via ERK activation. **a** Hematoxylin and eosin staining of teratomas derived from ChR2-GFP-V6.5 ESCs in both the photoactivated group and the nonphotoactivated control group. Ectoderm, mesoderm and endoderm are marked with different color arrows. **b** qRT-PCR analysis of the mRNA level of lineage-specific genes in photoactivated ChR2-GFP-V6.5 ESCs and nonphotoactivated control cells. **c** Western blot analysis of the total ERK and p-ERK in ChR2-GFP-V6.5 ESCs after different intensities of optical stimulation (low intensity: 0.1 mW, medium intensity: 1 mW, high intensity: 10 mW). **d** Statistical analysis of the expression levels of ERK and p-ERK in photoactivated and nonphotoactivated ChR2-GFP-V6.5 ESCs. **e** Immunostaining for the pluripotency markers OCT4, SSEA-1, SOX2 and NANOG in photoactivated, photoactivated with MEK inhibitor PD0325901 (1 mM), nonphotoactivated and nonphotoactivated with MEK inhibitor PD0325901 (1 mM) ChR2-GFP-V6.5 ESCs. **f** qRT-PCR analysis of the pluripotency-associated genes *Oct4, Sox2*, *Nanog*, *Esrrb* and *Klf4* in photoactivated, photoactivated with MEK inhibitor PD0325901 (1 mM), nonphotoactivated and nonphotoactivated with MEK inhibitor PD0325901 (1 mM) ChR2-GFP-V6.5 ESCs. **g** AP staining of in photoactivated, photoactivated with MEK inhibitor PD0325901 (1 mM), nonphotoactivated and nonphotoactivated with MEK inhibitor PD0325901 (1 mM) ChR2-GFP-V6.5 ESCs (n = 6, *P < 0.05, **P < 0.01, ***P < 0.001 compared to the nonphotoactivated control). **h** Summarize of calcium wave by photoactivation or KCl with or without extra-cellular calcium

The results above showed that photoactivation could substantially reduce the mRNA levels of TFs, including *Oct4*, *Nanog*, *Sox2*, *Esrrb* and *Klf4*. Moreover, teratomas derived from ChR2-GFP-V6.5 with photoactivated group largely appeared ectoderm structures, while endoderm was distinctly less than that of nonphotoactivated group (Fig. 6a). Next, to address whether blue light stimulation acts as a permissive signal to initiate ChR2-GFP-V6.5 ESC differentiation, we asked whether this stimulation could modulate the lineage by measuring the mRNA level of lineage-specific markers. Interestingly, photoactivation resulted in an increased mRNA level of various differentiation markers (including *Branchyury*, *Hand1*, *Fgf5*, *Cdx2* and *Nestin*), especially markers of the trophectoderm and ectoderm fates (*Cdx2*, *Fgf5* and *Nestin*) (Fig. 6b) (n = 6, P < 0.05 vs. control). ERK activation plays an important role in the initiation of ESC differentiation [20]. However, whether optical stimulation could affect intracellular ERK activity was unclear. Thus, we treated ChR2-GFP-V6.5 ESCs with different intensities of light stimulation for 48 h and analyzed the phosphorylation of ERK. Western blot analysis showed that after 48 h of photoactivation, the ERK activity was significantly upregulated as the light intensity increased (Fig. 6c, d) (n = 6, P < 0.05 vs. control). Furthermore, the MEK inhibitor PD0325901 could obviously rescue the down-regulated mRNA level and expression of core transcriptional factors OCT4, SSEA-1, SOX2 and NANOG in the photoactivated group partially (Fig. 6e, f) (n = 6, P < 0.05 vs. control). The application partially rescue the colony-form ability of ChR2-GFP V6.5 ESCs inhibited by photoactivation (Fig. 6g). PD0325901 has no obvious effect on the nonphotoactivated group (Fig. 6e–g) (n = 6, P < 0.05 vs. control). Furthermore, we have observed the calcium wave in the ChR2-GFP-V6.5 ESCs. We found the slow calcium wave without light stimulation, and the light stimulation could obviously elevate the calcium activity of ChR2-GFP-V6.5 ESCs. We also observed the calcium activity induced by KCl, which could induce membrane depolarization. Moreover, after elimination the extra-cellular calcium, we could still observe the light induced calcium activity (Fig. 6h).

## Discussion

In our present work, by applying optogenetics, we found that optical stimulation could not only inhibit the proliferation of ChR2-GFP-V6.5 ESCs but also induce the transition of ChR2-GFP-V6.5 ESCs from self-renewal to differentiation. Notably, we observed that optical stimulation enhanced the activation of the ERK signaling pathway, which is important for controlling mESC fate.

Optogenetics offers biologists a new way to stimulate cells [21]. ChR2 has been extensively used to explore neural circuits because it provides precise temporal and spatial information while being noninvasive [22, 23]. Interestingly, utilizing light to control signaling pathways is an attractive approach in cell biology, as low intensity light can activate cells with temporal precision and no damage [24]. ESCs hold great promise for regenerative medicine as these cells can indefinitely propagate and generate any of the specialized cell types [25]. In addition, ESCs can be used to study the development and function of human tissues [26]. The stemness state is controlled by the core TFs including *Oct4*, *Sox2*, and *Nanog* [6, 27–30], and ESC differentiation involves inhibition of the core TFs, resulting in the activation of lineage-specific genes [31, 32]. However, the intrinsic mechanism controlling the pluripotent state and initiation of differentiation needs more study [33]. Many signaling pathways involved in mESC self-renewal and differentiation have been identified by applying agonists and antagonists. However, methods facilitating the precise control of cell signaling have also been intensely explored [34].

mESCs expressing ChR2 were used to analyze the integration of grafted neurons at synaptic level in the neural system in vitro [14–16]. We chose a much higher light density (5 min/h, 10 mW/mm^2^) to stimulate the mESCs. Under these conditions, we found that optical stimulation-induced depolarization inhibited ChR2-GFP-V6.5 ESC proliferation and self-renewal and initiated the differentiation of ChR2-GFP-V6.5 ESCs without affecting the cell viability. Changes in membrane potential could affect the fate decision of ESCs, as the membrane became hyperpolarized when the ESC cell cycle progressed from the G_1_ to the S phase but became depolarized when the ESCs started to differentiate [35]. When the K^+^ channel expressed on ESCs was blocked, the proliferation and cell cycle progression of ESCs were significantly inhibited [8, 36]. RA, an agent that is essential for embryonic neural development, could enhance differentiation by inducing membrane depolarization [37], and an endogenous RA gradient has been detected during embryonic development via a live imaging method [38]. In our study, after optical stimulation, the proliferation rate of mESCs was reduced, and the cell cycle was inhibited. As ChR2 channels that are opened by blue light exposure depolarize the cell [23, 39], it is possible that the normal membrane potential oscillations of mESCs were disrupted by light stimulation, leading to differentiation of the mESCs. However, light stimulation on mESC did not lead to differentiation to a specific lineage.

We detected activation of the ERK signaling pathway, which is involved in the inhibition of ChR2-GFP-V6.5 ESC proliferation and initiation of ChR2-GFP-V6.5 ESC differentiation, after optical stimulation, and the activation occurred in a light intensity-dependent manner. ERK activity could regulate ESC self-renewal and fate determination [40, 41]. Inhibition of ERK activity promotes mESC self-renewal by regulating core TFs, such as Nanog [29, 42–44]. Moreover, the differentiation of ESCs could be blocked by an ERK inhibitor [45], and glycogen synthase kinase 3 (GSK3) could maintain mESC long-term pluripotency without Leukemia inhibitory factor (LIF) [41, 46]. Bone morphogenetic protein (BMP) controls the ESC fate decision by tuning ERK activity through DUSP9 [47]. Furthermore, activation of ERK triggered the transition of ESCs from self-renewal to differentiation [20]. The FGF/MEK/ERK pathway is essential for trophectoderm and primitive endoderm formation in murine embryonic development [44, 48–50]. The ERK signaling pathway regulates neuroectoderm specification via the regulation of Poly (ADP-ribose) polymerase 1 (PARP-1) activity in mESCs [51–53].

Therefore, in our study, the differentiation of ChR2-GFP-V6.5 ESCs after optical stimulation might occur due to the activation of the ERK signaling pathway. However, the MEK inhibitor PD0325901 could partially rescue the down-regulated expression of core transcriptional factors induced by optical stimulation. Many extracellular factors could affect the stem cell state [54]. Thus, we do not rule out a potential role of other signaling pathways in the ChR2-GFP-V6.5 ESC differentiation induced by photoactivation. For instance, similar to ERK signaling, the calcineurin-NFAT-Src pathway triggers mESC differentiation. These two distinct signaling pathways might cooperate to control mESC fate [55]. Previous studies have also found that optical stimulation could induce an influx of Ca^2+^ into the cytoplasm [56]. Intracellular Ca^2+^ regulates the proliferation, apoptosis and differentiation of cells through activating downstream pathways [57]. We have observed the increase of intra-cellular calcium concentration after 470 nm light stimulation. In future studies, we will put particular emphasis on the role of Ca^2+^ and its downstream signaling pathways in the initiation of differentiation after photoactivation. Further study might provide a new understanding of the initiation of the differentiation of ChR2-GFP-V6.5 ESCs after photoactivation.

In conclusion, by utilizing optogenetic techniques, we have demonstrated that cell membrane depolarization plays an important role in modulating the proliferation, self-renewal and initiation of differentiation of mESCs. Our results demonstrate that the activation of ERK is involved in the transition of mESCs from self-renewal to differentiation that is induced by membrane depolarization.
